# Technology-dependency among patients discharged from a children's hospital: a retrospective cohort study

**DOI:** 10.1186/1471-2431-5-8

**Published:** 2005-05-09

**Authors:** Chris Feudtner, Nanci Larter Villareale, Barbara Morray, Virginia Sharp, Ross M Hays, John M Neff

**Affiliations:** 1 Pediatric Advanced Care Team, Integrated Care Service, and Pediatric Generalist Research Group, The Children's Hospital of Philadelphia; Philadelphia, USA; 2 The Leonard Davis Institute and the Center for Bioethics, University of Pennsylvania, Philadelphia, USA; 3 Center for Children with Special Needs, Children's Hospital & Regional Medical Center, Seattle, USA; 4 Pediatric Palliative Care Program, Children's Hospital and Regional Medical Center, Seattle, USA; 5 Department of Rehabilitation Medicine, University of Washington, Seattle, USA; 6 Department of Pediatrics, University of Washington, Seattle, USA

## Abstract

**Background:**

Advances in medical technology may be increasing the population of children who are technology-dependent (TD). We assessed the proportion of children discharged from a children's hospital who are judged to be TD, and determined the most common devices and number of prescription medications at the time of discharge.

**Methods:**

Chart review of 100 randomly selected patients from all services discharged from a children's hospital during the year 2000. Data were reviewed independently by 4 investigators who classified the cases as TD if the failure or withdrawal of the technology would likely have adverse health consequences sufficient to require hospitalization. Only those cases where 3 or 4 raters agreed were classified as TD.

**Results:**

Among the 100 randomly sampled patients, the median age was 7 years (range: 1 day to 24 years old), 52% were male, 86% primarily spoke English, and 54% were privately insured. The median length of stay was 3 days (range: 1 to 103 days). No diagnosis accounted for more than 5% of cases. 41% were deemed to be technology dependent, with 20% dependent upon devices, 32% dependent upon medications, and 11% dependent upon both devices and medications. Devices at the time of discharge included gastrostomy and jejeunostomy tubes (10%), central venous catheters (7%), and tracheotomies (1%). The median number of prescription medications was 2 (range: 0–13), with 12% of cases having 5 or more medications. Home care services were planned for 7% of cases.

**Conclusion:**

Technology-dependency is common among children discharged from a children's hospital.

## Background

What proportion of children discharged from children's hospitals are technology-dependent (TD)? Advances in medical technology over the past century have profoundly changed the landscape of pediatric ambulatory and hospital care [1-3]. Through the combined effects of pharmaceuticals and medical devices, parents and health care professionals now care for a population of children who depend on medical technology to live or remain in their current state of health.

TD children and the complexity and costs of their care gained widespread attention in the United States during the 1980s. In a November 1981 news conference President Reagan cited the case of Katie Beckett, a 3-year-old Iowa girl who (due to lack of any means to fund home care) had lived in a hospital since having viral encephalitis at 3 months of age [4]. Two days later, a special waiver was issued for Ms. Beckett's case [5], and by the following summer the Secretary of the US Department of Health and Human Services had established a waiver program that enabled an individual residing at home to continue to be covered by Medicaid and receive Medicaid-funded long term care services [6]. In December 1982, the US Surgeon General sponsored a conference that focused on the example of "the ventilator-dependent child" [7]. Then in 1984 the US Supreme Court ruled that a child with spina bifida who required clean intermittent catheterization had the right to receive such a service in the school setting in order to benefit from special education [8].

**Figure 1:**
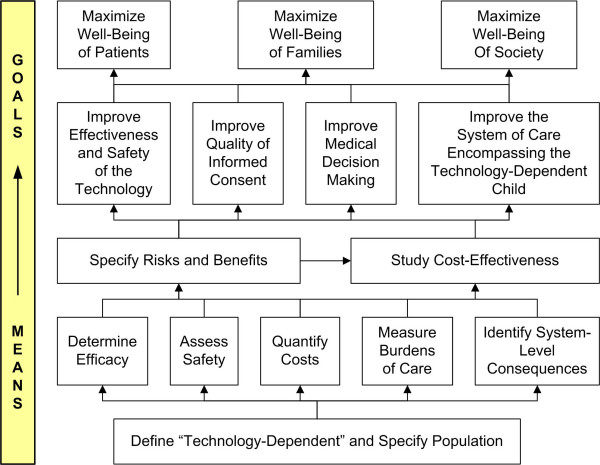
Goal and means for the understanding of technology-dependency.

**Figure 2:**
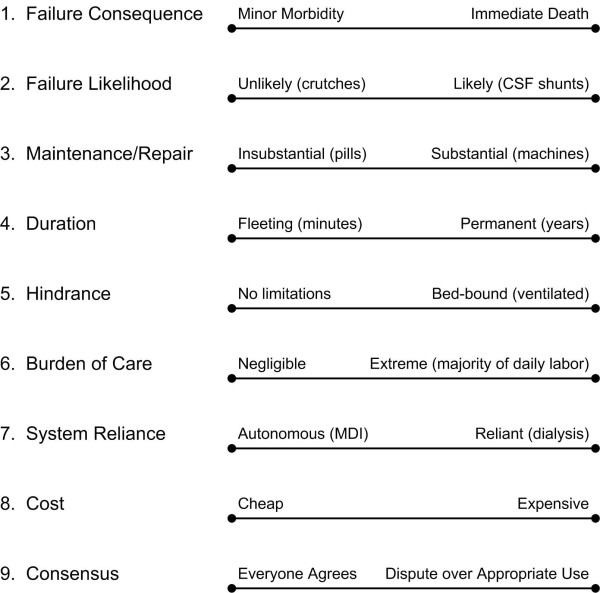
**Dimensions of dependency on technology. **Examples enclosed in parentheses are meant only to illustrate extremes of the dimensions. CSF, cerebrospinal fluid; MDI, metered dose inhaler.

**Figure 3:**
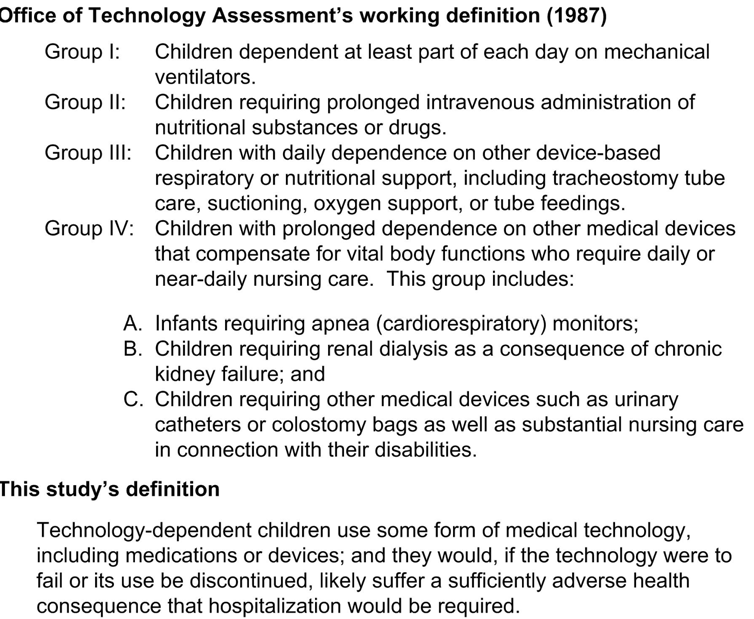
Definitions of technology-dependent.

The well-being of TD children, and the effect that caring for these children has on the well-being of their families and of society, is an important concern for clinicians, ethicists, and healthcare policy analysts [9]. The goals of maximizing the well-being of TD children, their families, and society (Figure 1) are supported by information generated by various studies such as an evaluation of pediatric home-ventilator programs [10]; cross-sectional observational population-based epidemiologic studies documenting the prevalence of technology-dependent children [11-14]; and surveys focusing on the health, psychosocial, and financial impact on mothers, parents, and families [15-23]. Given that dependence on technology is a complex multidimensional construct (Figure 2), each of these studies has had to define "technology-dependent," a task that has fundamental implications for the ensuing research. The most commonly cited definition of TD originated in a 1987 report issued by the former federal Office of Technology Assessment (OTA), which proposed as a "working definition" the scheme outlined in Figure 3[11]. The OTA report underscored how their hierarchical definition reflected both consensus expert opinion that OTA groups I-III were technology-dependent and the government's desire to limit expenditures by excluding OTA group IV from the definition, since the prevalence of children meeting the group IV definition was so large.

Our research group, in an effort to improve the quality of services offered to hospitalized technology-dependent children and their families, developed a definition of TD children that emphasizes the failure consequence dimension of the TD construct. As indicated in Figure 3, we specified children as TD if the failure or cessation of a device or drug that they were using would have the likely consequence of a required hospitalization. While our definition does not explicitly encompass other dimensions of the TD construct, hospitalization would also be indicative of greater hindrance, system-reliance, and cost. Of note, our definition did not specify a minimal duration of dependency, since from a quality-improvement perspective the duration of dependency is not necessarily the most important dimension of the underlying TD construct. Although most studies of TD have focused only on reliance on machines or devices, we also considered medications as a form of technology, in keeping with the definition of technology put forth in the OTA report [11], and in keeping with our underlying concern regarding the effectiveness, safety, and burden of medical interventions that persist beyond the period of hospitalization.

Guided by this definition, we sought to determine the proportion of children discharged from a tertiary care children's hospital who are TD, describe their characteristics, and examine aspects of the care provided to these children upon discharge.

## Methods

After obtaining Human Subjects Research Committee approval, we conducted a retrospective cohort study of a random sample of 100 patients admitted to Children's Hospital and Regional Medical Center in Seattle, Washington, a regional tertiary care center with approximately 11,000 hospitalizations annually. Electronic administrative data regarding all discharges during calendar year 2000 was used to select the subjects using a random number table. Eligible subjects included all pediatric patients admitted to any hospital service and of any age; adults admitted for the purpose of tissue or organ donation to a pediatric patient were excluded. For subjects who were discharged more than once during 2000, we randomly selected a single hospitalization episode. No patient was randomly selected twice.

For all 100 randomly-selected eligible hospitalizations of 100 patients, two investigators conducted a structured hospitalization chart review and abstraction using a data collection instrument. Prior to the conduct of this study, we had developed this data collection instrument specifically for this project, and pilot tested the instrument by having both investigators independently abstracting the same series of 5 hospitalization records and refining the instrument to assure consistent agreement regarding the abstracted data. Data collected included demographic information, all documented diagnoses, procedures, technology use, medication orders, feeding orders, and nursing orders, as well as whether home nursing services were ordered for post-discharge care.

Four investigators, consisting of three pediatricians and one nurse with a range of 6 to 40 years experience caring for children with special health care needs, then independently reviewed all 100 chart abstractions and determined whether each subject at the time of discharge from the hospital met the definition specified in Figure 3. We also sought to situate the construct of TD within the construct of children with special health care needs (CSHCN), which has been defined by the Maternal and Child Health Bureau of the United States as: "All children who have, or are at increased risk for, chronic physical, developmental, behavioral, or emotional conditions and who also require health and related services of a type or amount beyond that required by children generally." The MCHB definition is further refined by the definition of "chronic" as conditions expected to last 6 months or longer.

Given limitations in the data available in the hospitalization record and the nature of the TD and CSHCN definitions, the 4 investigators had to employ their knowledge and judgment to make the determination of TD or CSHCN. To assess the degree of consensus in these judgments, we used a 4-rater kappa statistic to compare the four evaluators' independent dichotomous ("yes or no") classifications of each subject regarding TD (kappa = 0.56) and CSHCN (kappa = 0.59). These values for the 4-rater kappa indicate moderate verging on good agreement. For the subsequent analysis of the data, only those cases where 3 or 4 raters independently classified the subject as either TD or CSHCN were considered to be TD or CSHCN. Examples of cases where only 2 of the 4 investigators interpreted the chart abstract data as indicated TD (and thus were not considered TD for the purposes of this study) included: a patient with the diagnoses of a brain tumor and obstructive hydrocephalus but no documented evidence of a cerebrospinal fluid shunt; a patient with a diagnosis of a malignancy but no documentation of discharge medications; and several instances of patients diagnosed with apparent persistent asthma but no documentation of a controlled medication having been prescribed at the time of discharge.

Characteristics of the sample subjects were then examined by calculating medians, ranges, and proportions. Inferences drawn from this random sample regarding the composition of the complete source population (that is, all children discharged from this hospital) were expressed as 95% confidence intervals of the sample proportions, derived from the binomial distribution. All analyses were conducted using Stata 8.2 (StataCorp, College Station, TX).

## Results

Within our random sample of 100 patients admitted to a regional pediatric tertiary care center (Table 1), the mean age was 7.6 years (median 7.1 years). Just over half (52%) of the subjects were male. The majority (86%) were identified as speaking English as their primary language, and 54% as covered by private insurance.

**Table 1:** Demographic characteristic of subjects

		Percentage (%) (n = 100)	95% CI
Age			
	Less than 1 month	7	3 – 14
	1 – 11 months	15	9 – 24
	1 – 4 years	21	13 – 30
	5 – 9 years	21	13 – 30
	10 – 14 years	24	16 – 34
	15 – 24 years	12	6 – 20
Gender			
	Male	52	42 – 62
	Female	48	38 – 58
Language			
	English	86	78 – 92
	Spanish	7	3 – 14
	Other	3	0.1 – 9
	Unknown	4	0.1 – 10
Principal Insurance			
	Private	54	44 – 64
	Government	40	30 – 50
	Self-insured or none	4	1 – 10
	Unknown	2	0.2 – 7

The primary reasons for hospitalization (Table 2) were led by cancer and the treatment of cancer (15%), followed by respiratory infections (9%), asthma (5%), gastroenteritis (5%), appendicitis (4%), epilepsy or seizures (3%), and the malfunction of a device or graft (3%). Most patients stayed 3 days or less (59%), although 14% of subjects were hospitalized 8 days or more (maximum of 103 days) during this hospitalization. Most subjects were discharged to home (91%), one subject died during the hospitalization, and 3% were transferred to another facility. Home nursing care was arranged for 8% of the subjects, and 7% had other forms of home care ordered.

**Table 2:** Medical characteristics of subjects

		Percentage (%) (n = 100)	95% CI
Most frequent diagnostic categories *			
	Neoplasm and chemotherapy	15	9 – 24
	Respiratory infections	9	4 – 16
	Asthma	5	2 – 11
	Gastroenteritis	5	2 – 11
	Appendicitis	4	1 – 10
	Epilepsy or seizure	3	0.6 – 9
	Malfunctioning device or graft	3	0.6 – 9
Length of stay			
	1 day	10	5 – 18
	2 days	25	17 – 35
	3 days	24	16 – 34
	4–7 days	27	17 – 37
	8 to 103 days	14	8 – 22
Devices *			
	Gastrostomy or jejuenostomy tubes	10	5 – 18
	Central venous line	7	3 – 14
	Medication nebulizer	7	3 – 14
	Ventriculoperitoneal shunt	2	0.2 – 7
	Tracheotomy	1	0.03 – 5
Number of medications §			
	0	18	11 – 27
	1	20	13 – 29
	2	26	18 – 36
	3	16	10 – 26
	4	7	3 – 14
	5 or more	12	6 – 20
Number of daily medication administrations noted ‡			
	0 or PRN	33	24 – 44
	1–4	30	21 – 40
	5–9	24	16 – 34
	10–22	12	6 – 20
Home nursing care		8	4 – 15
Other forms of home care		7	3 – 14
Disposition			
	Home	91	84 – 96
	Transfer	3	0.6 – 9
	Death	1	0.03 – 6
	Unknown	5	2 – 11

* For the categories of diagnoses and devices, a given subject could be counted multiple times if noted to have had multiple diagnoses or devices. §Medications were counted as those medications ordered at the time of discharge from the hospital; accordingly, the one case that died is omitted so that n = 99 for this variable as well as "number of daily medication administrations". ‡ Number of daily medication administrations is a count of the number of times any medication dose is delivered in a day, so that a count of 10 could indicate 2 medications given 5 times a day, or 10 medications each given once.

A quarter of all subjects (26%) used some form of medical device (Table 2), including gastrostomy or jejenonostomy tubes (10%), central venous catheters (7%), medication nebulizer (7%), ventriculoperitoneal cerebrospinal fluid shunts (2%), or tracheotomies (1%). Only 18% of subjects were discharged from the hospital with no medications ordered, whereas the majority of subjects (61%) had 2 or more prescriptions ordered, and 12% had 5 or more medications ordered. Focusing on one parameter that contributes to the burden of care – namely, the number of times each day that any form of any medication was ordered to be administered – 12% of subjects were ordered at the time of discharge to have between 10 and 22 daily medication administrations.

**Figure 4:**
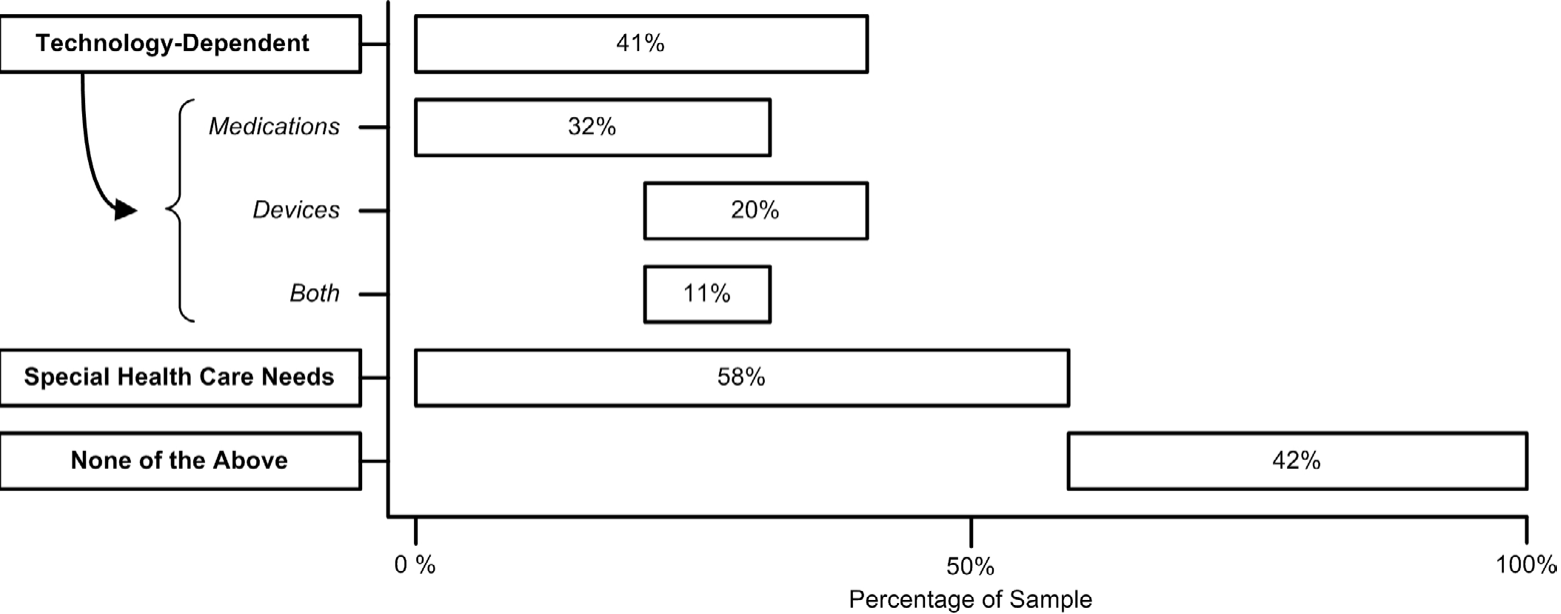
**Proportion of subjects with technology dependency or special health care needs. **The span of each bar represents the proportion of the sample that exhibited the specified characteristic. The overlapping position of the bars vis-à-vis each other represents the proportion of subjects who exhibited several of these characterizes. *Medications*, *Devices*, and *Both *(medications and devices) indicate sub-types of technology-dependence.

On the basis of information contained in the hospitalization record, 41% of all subjects were judged as being dependent on some form of technology, specifically 20% being dependent upon medical devices, 32% being dependent upon medications, and 11% being dependent upon both medical devices and medications (Figure 4). Among the subjects deemed to be dependent upon medications, the most prevalent clinical circumstances were children receiving either anti-epileptic drugs, long-term anti-inflammatory therapy, or anti-neoplastic and related medications, with other children dependent upon insulin, long-term analgesic medications, or anti-gastrointestinal reflux drugs. Fifty-eight percent of all subjects were deemed to meet the MCHB criteria of children having special health care needs.

TD patients were older than non-TD patients (median age category 10–14 years versus 1–4 years, respectively, p = 0.01), and were less likely to be covered by private insurance than non-TD patients (43.9% versus 61%, p = 0.06). TD patients were discharged with a greater number of prescriptions than patients without TD (mean 4.1 versus 1.3, p < 000.1, with the respective ranges being 0 to 15 versus 0 to 3). Overall, TD patients were not more likely to receive home services than non-TD patients (14.6% versus 8.5%, p = 0.3), but those patients dependent upon devices were more likely to receive home care than patients without device dependency (25% versus 7.5%, p = 0.03).

## Discussion

This detailed review of 100 randomly selected patients admitted to a regional pediatric tertiary care center in the year 2000 suggests that a sizable proportion of all admitted patients (41%) are substantially dependent upon some form of medical technology in order to avoid deterioration in health that would necessitate hospital admission.

Our findings need to be interpreted in the context of this study's limitations. First, the degree to which the results can be generalized beyond the year 2000 for this single institution, serving as a tertiary referral center for a multi-state region in the Pacific Northwest, is unclear. An analysis of pediatric hospitalization time-trends in Washington State revealed that the care of children with complex medical conditions increasingly is concentrated in pediatric referral centers [24], suggesting that TD children may compose an enlarging proportion of discharges from children's hospitals. Second, the study's random sample of 100 distinct child discharges, while providing representative estimates of characteristics of all patients discharged from this hospital, is not as precise as a larger sample would be. For example, the small sample likely explains why certain technologies such as oxygen therapy, ventilator support, or renal dialysis were not observed. Third, the classification of patients into the categories of TD or CSHCN required the use of individual raters' judgment. While agreement among the 4 raters was moderate to good, we only classified patients into these categories if 3 of 4 raters agreed, thereby improving specificity but at the loss of sensitivity, so that our data may underestimate the proportions of both TD and CSHCN children. Finally, our study measures only a few attributes of care that can make care burdensome; the study also has no information regarding post-discharge occurrences, such as adherence to discharge instructions, the adequacy of follow-up ambulatory care, or unintended hospital readmissions.

These limitations notwithstanding, the prevalence of TD in this population of hospitalized children suggests that the phenomenon of technology-dependency warrants further study. As suggested by Figure 2, research efforts could seek a) to advance the performance of the technology, b) to assist families and care providers in making better decisions about when to adopt specific technologic solutions to health problems, and c) to improve how the encompassing system of health and supportive care maximizes the benefits of the technology while minimizing the associated risks and burdens. Accomplishing these aims through risk/benefit, cost/effectiveness, and cost/utility analyses will require more precise quantification of technologies' benefits, risks, costs, burdens, and broader societal consequences.

Three specific challenges hinder the pursuit of such a research agenda. First, no consensus definition of "technology-dependency" currently exists, either as a yes/no or as gradations of a technology-dependency classification scheme, perhaps based on the multidimensional construct proposed in Figure 2. The merits of any particular definition used to classify children as TD should be viewed in light of whether the definition can be used readily, reliably, accurately, and consistently for research purposes; and perhaps most important, whether the definition when applied enables the subsequent analysis to advance a value-focused research agenda that ultimately maximizes patient, family, and public well-being. Second, methods to assess the burden of care, quantitatively as well as qualitatively, need to be developed. Lacking such measures, evaluations of technology are likely to underestimate the indirect costs borne by families and care providers, and broader consequences to society. Third, better assessment of the value that children, parents, and others place on TD quality of life – and how these evaluations change over time with increased exposure to the technology and the quality of life that it supports – is essential not only for cost-utility analyses, but also to enable children or parents to make better informed decisions when presented with TD options of care.

Addressing these challenges would enable 1) documentation of technology-dependency incidence and changes in prevalence over time; 2) assessment of the impact of technology-dependency on patients' and families' physical, psychosocial, and financial well-being throughout an illness trajectory; 3) identification of health-care providers and agencies that care for TD children and the economic ramifications of providing such care; 4) evaluation of how TD patients and the care that they require affects the health-care system as well as schools, other social services, and parental employers; and 5) testing of specific interventions and management techniques that conceivably influence the burden of TD care. With the information that such studies would provide, we would be better able to perform individual- and population-level needs assessment and planning, to develop techniques to lessen burdens and increase the safety and efficacy of TD, and to create policies to promote high-quality care for these vulnerable patients and their families.

## Conclusion

A substantial proportion of children discharged from a regional children's hospital were dependent upon technology. Further study is required to ascertain the quality of care received by TD children and their families and then potentially to improve their outcomes.

## Competing interests

The author(s) declare that they have no competing interests.

## Authors' contributions

All authors participated in the design of the study and interpretation of the data; NLV and BM conducted the chart reviews; CF and VS performed the data analysis; CF drafted the manuscript; all authors revised the manuscript for key intellectual content. All authors read and approved the final manuscript.

## Pre-publication history

The pre-publication history for this paper can be accessed here:


